# Impact of Hypothermia and Oxygen Deprivation on the Cytoskeleton in Organ Preservation Models

**DOI:** 10.1155/2018/8926724

**Published:** 2018-07-16

**Authors:** Raphael Thuillier, Thierry Hauet

**Affiliations:** ^1^CHU Poitiers, Service de Biochimie, Poitiers 86000, France; ^2^Inserm U1082, Poitiers 86000, France; ^3^Université de Poitiers, Faculté de Médecine et de Pharmacie, Poitiers 86000, France; ^4^Fédération Hospitalo-Universitaire SUPORT, Poitiers 86000, France; ^5^IBiSA Plateforme “MOPICT”, Institut National de la Recherche Agronomique, Unité Expérimentale Génétique, Expérimentations et Systèmes Innovants, Domaine Expérimental du Magneraud, Surgères 17700, France

## Abstract

Ischemia reperfusion (IR) lesions are an unavoidable consequence of organ transplantation. Researching new therapeutics against these lesions requires the definition of early mechanisms. The cytoskeleton is composed of 3 types of filaments: microfilaments, intermediate filaments, and microtubules. We aimed to characterize the influence of preservation on their phenotype. In an* in vitro* model using primary human endothelial cells reproducing the conditions of organ preservation, two aspects were explored: (a) the impact of IR and cold ischemia time on each filament type, evaluating the roles of temperature, solution, and oxygen; and (b) the potential of cytoskeleton-mediated therapy to alleviate cell death. Results showed that intermediary filaments were unaffected, while microfilaments showed radical changes with disappearance of the structure replaced by a disorganized array of nodules; moreover, microtubules almost completely disappeared with time. Furthermore, temperature, and not oxygen deprivation or the solution, was the determining factor of the cytoskeleton's loss of integrity during preservation. Finally, pharmaceutical intervention could indeed preserve fiber structure but did not alter survival. Our work shows that improvement of preservation must include a more adapted temperature before considering oxygen, as it could profoundly improve cytoskeleton organization and thus cell fate. This highlights the importance of this structure for the development of new therapeutics and the definition of graft quality biomarkers.

## 1. Introduction

Chronic kidney disease prevalence in the US population now reaches 14% [1]. With a large amount of these patients evolving towards end stage renal disease, the need for organs to be transplanted, the most adapted treatment is constantly increasing. Organ demand is now fourfold higher than the donation rate, leading procurement organizations to extend donor criteria [2], to the detriment of organ quality. These suboptimal organs are more sensitive to ischemia reperfusion injury (IRI) [3], partly explaining the increased rate of short-term [4–6] and long-term [7, 8] complications. In this ongoing donor demographic change, it is now pivotal to better understand IRI mechanisms in order to design better preservation protocols and organ quality biomarkers.

Cell function is intimately related to its structure and thus to the integrity of this network composed of three types of fibers: (a) microfilaments of actin, responsible for cell shape and movement (contraction, crawling), as well as the intracellular movement of organelles; (b) intermediate filaments, responsible for cell shape and anchorage of organelles; and (c) microtubules, composed of *α*- and *β*-tubulin dimers, in charge of cellular movement (flagella, cilia), chromosome movement during division, and organelle movement and particularly of vesicular trafficking. Studies on the influence of preservation on the cytoskeleton have been conducted. In pulmonary endothelial cells, hypothermia induced actin and tubulin filaments disorganization [9]. In cultured hepatocytes, hypothermia induced microfilament precipitation and microtubule shortening [10]. In kidney epithelial cells, similar conditions caused disorganized microtubules while leaving intact the microfilaments, with a particular sensitivity of the proximal tubule cells [11]. Finally, in isolated kidney tubules hypothermia coupled to hypoxia induced a dysfunction of the microfilament-membrane interface and depolymerized microtubules [12]. However, the conditions in some of these studies were very different to those of organ preservation, regarding oxygenation, cold ischemia time (reaching days for some studies), and preservation solution. Hence, these conclusions are sometimes contradictory and difficult to apply to organ transplantation-related IRI.

Herein, we focused on the endothelium, due to its ubiquitous presence in all transplanted organs and the major role played as interface between the organ and the organism, particularly its immune system. Endothelial cells are indeed the principal regulators of the vasculature, in constant interaction with the blood and component of the vessel wall [13]. Moreover, they play a central role in the regulation of the inflammatory response, itself at the heart of several pathologies, including IRI [14]. The mechanisms underlying the hypothermia-induced injury on endothelial cells have only been partly resolved [15]; they include nuclear deformation, loss of adhesion, and cell death. Recently, mild hypothermia was shown to increase apoptosis resistance and reduce inflammatory cytokines production, except IL6 which was upregulated [16]. However, our own results show that organ preservation level hypothermia induces endothelial cell activation and a proinflammatory phenotype [8, 17].

In the present study, we determined the influence of organ preservation conditions on endothelial cells cytoskeleton, following each of the three subtypes of filaments during storage, as well as attempting to identify the individual influence of each of the three parameters altered during preservation: temperature, oxygenation, and solution.

## 2. Results

### 2.1. Evolution of Cytoskeletal Filament Organization during Cold Ischemia Time

#### 2.1.1. Microfilaments (Figure 1)

Actin staining was performed at different times during cold storage of endothelial cells. Results showed that control cells displayed regular and well organized stress fibers. However, as early as 6 hours after the start of hypothermic/hypoxic storage in UW, signs of disorganization appeared, with amorphous nodules of actin in the cytoplasm and shortened stress fibers. These features appeared more frequently with the elongation of cold ischemia time, leading to a complete loss of fibers within the cytoplasm as well as a peculiar organization at the cellular perimeter, in which short fibers appear to strike out of the cell.

#### 2.1.2. Microtubules (Figure 2)

*α* and *β* tubulin staining on control cells revealed a concentration of signal near the nuclei (centrosome region) and well-defined filaments reaching towards the extremity of the cell. Six hours after the start of storage, the signal was more diffuse with less defined filament. At 12h, the signal intensity was decreased, with diffuse staining in the perinuclear region and appearance of dot-like staining at the extremities of the cell instead of fibers. Further time points revealed a similar staining pattern, albeit with decreased intensity.

#### 2.1.3. Intermediate Filaments (Supplementary Figure 1)

Vimentin was the main protein for these filaments in our cells. Staining showed that, in control cells, the filaments were organized in a clearly defined network within the cell, with concentrated staining near the nuclei. Unlike the other filament subtypes however, cold storage did not appear to affect the staining pattern, at any time point during the 24h period.

### 2.2. Impact of Cold Ischemia on Cytoskeleton Proteins

**Figure 3:**
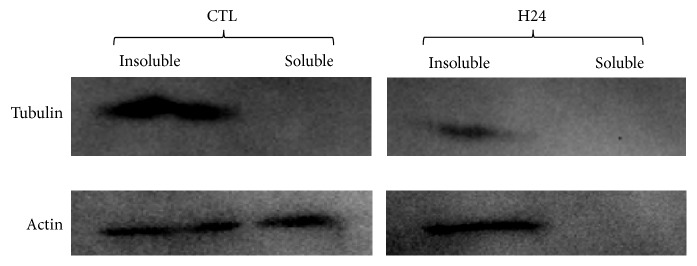
**Western blot analysis of cytoskeleton proteins during cold ischemia**. HAEC were cultured in hypoxia/hypothermia using UW solution for 24h; then the soluble and insoluble protein fractions were separated as per the Materials and Methods.

To uncover the mechanism underlying the observed rearrangement in tubulin and actin structure, we performed a separation of cell proteins permitting us to differentiate between soluble proteins (i.e., not attached to the cytoskeleton) and insoluble proteins (Figure 3).

Tubulin probing (Figure 3 (top)) showed that the majority of the protein was found in the insoluble fraction in the control cells, indicating that the majority of *α* and *β* tubulin were polymerized. After 24h cold ischemia, we observed a reduction of the amount of protein present in the insoluble fraction, while there was no change in the soluble fraction. This indicated that the phenotype observed by immunocytochemistry was likely due to a decrease of the total tubulin present, either through degradation or arrest of production.

We then detected *β* actin on the membranes (Figure 3 (bottom)), showing that in control cells there was protein in both fraction, with a higher amount present in the insoluble fraction. This was concordant with the constant renewal of actin microfilament, which requires a constantly rotating pool of nonpolymerized proteins. However, in the cells subjected to 24h cold ischemia, all proteins were present in the insoluble fraction, with no significant change in the amount of total actin.

### 2.3. Individual Role of Solution, Temperature, and Oxygen in the Cytoskeleton

**Figure 4:**
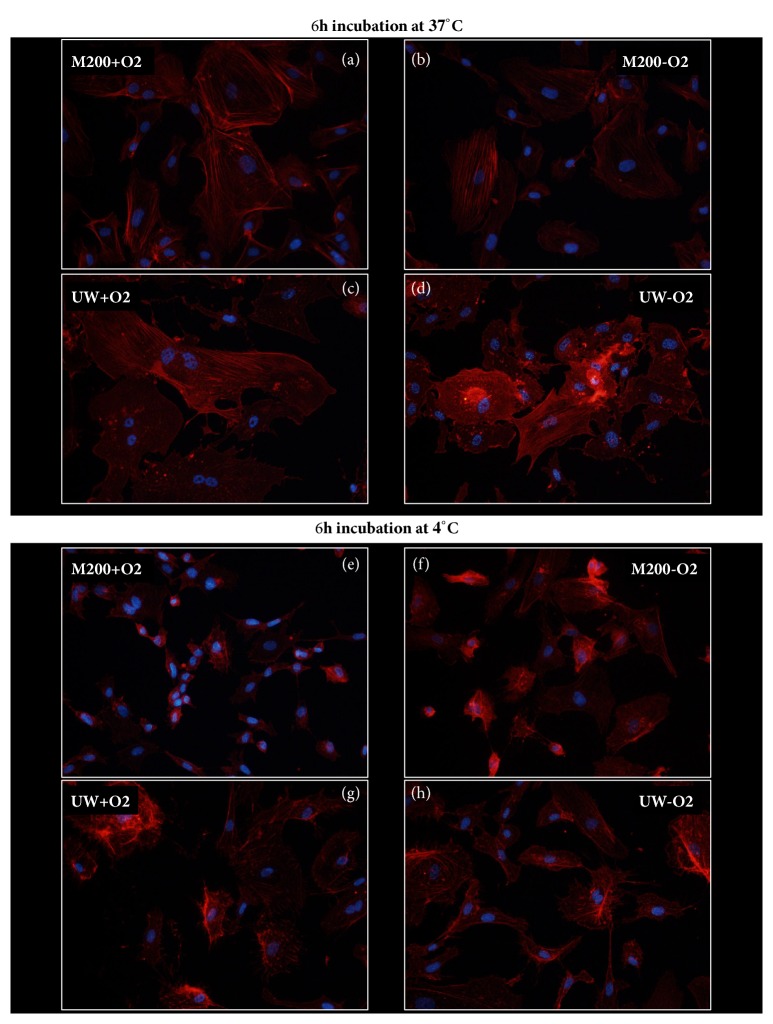
**Microfilament phenotype alteration after 24h: influence of solution, temperature, and oxygenation level**. HAEC were cultured in different conditions for 24h and then stained with phalloidin as per the Materials and Methods. Representative staining is shown for each condition.

**Figure 5:**
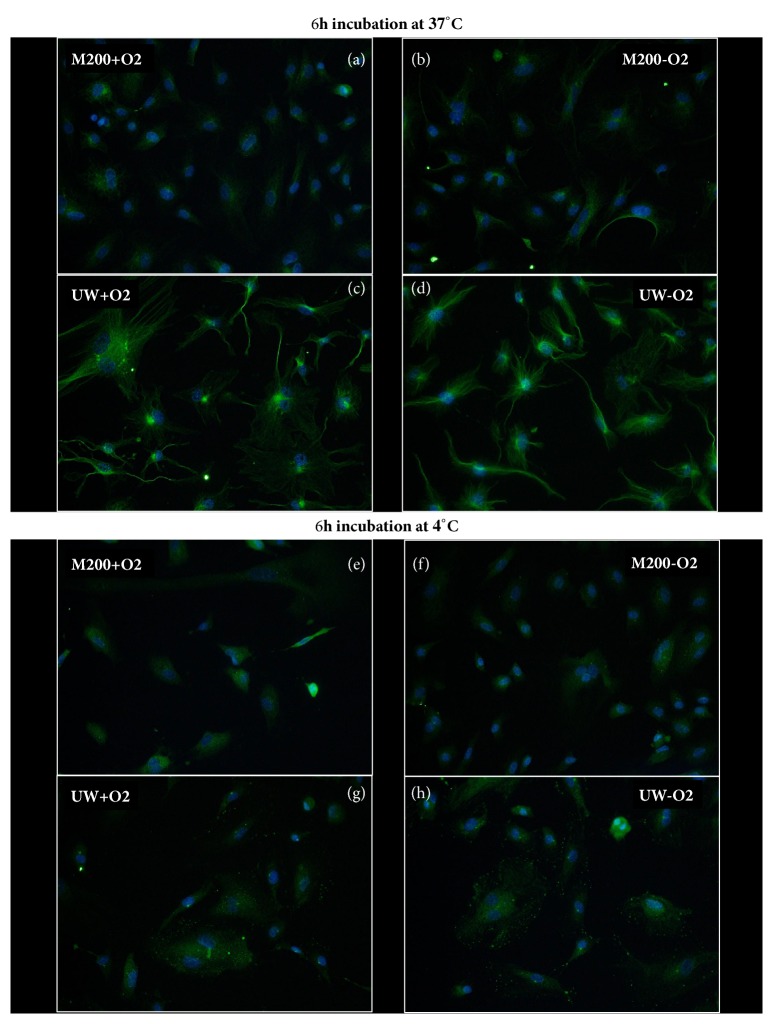
**Microtubules phenotype alteration after 24h: influence of solution, temperature, and oxygenation level**. HAEC were cultured in different conditions for 24h and then stained with an anti *α* and *β* tubulin antibody as per the Materials and Methods. Representative staining is shown for each condition.

To further understand the impact of cold ischemia on the cytoskeleton, we attempted to study the influence of each variable individually. In these figures (Figures 4 and 5 as well as Supplementary Figures 2–5) culture at 37°C in M200 with oxygen was the control condition: the condition in which these cells were cultured in the incubator, in accordance with the supplier's recommendations. Although intermediate filaments did not appear altered by the protocol, we investigated the effect of each parameter on vimentin staining (Supplementary Figures 4 and 5). In accordance with previous findings, no alterations were observed in this staining, with the exception of UW preserved cells at 37°C; however, these are likely due to the use of UW at 37°C.

#### 2.3.1. Solution Effect

Indeed, the inadequacy of UW to maintain cell integrity at 37°C was evidenced as early as 6 hours after the start of the experiment (Pictures (c) and (d) of Figures 4 and 5 as well as Supplementary Figures 2, 3, 4, and 5). Hence, we were only able to compare the solution effect on the cells at 4°C.

Regarding microfilaments, at 6h the level of alterations in M200 appeared higher than in UW preserved cells (Figure 4, (e) and (f) versus (g) and (h)), as the later displayed more prominent stress fibers and appeared more spread out on the culture surface. However, these differences were not found after 24h of preservation (Supplementary Figure 2, E and F versus G and H).

Concerning microtubules, we observed different phenotypes in cells preserved with the two solutions after 6h (Figure 5, (e) and (f) versus (g) and (h)): UW preserved cells displayed decreased tubulin staining around the cells nuclei and dot-like signal towards the extremities; however, M200 cells showed an even reduced signal, with only faint tubulin staining in the perinuclear region. At 24h, the staining patterns were similar between conditions.

#### 2.3.2. Temperature Effect

Since UW did not permit cell survival at 37°C, the only acceptable solution to be used for proper comparison of temperature was M200.

Regarding microfilaments, we confirmed the high degree of disturbance found in filament organization caused by hypothermia regardless of oxygen levels at both 6 (Figure 4(b) versus (f)) and 24 hours (Supplementary Figure 2).

Microtubule organization was again severely disturbed at both 6 (Figure 5  (b) versus (f)) and 24 hours (Supplementary Figure 3) by hypothermia.

#### 2.3.3. Oxygen Effect

Microfilaments staining revealed little to no difference between cells preserved in the absence or in the presence of oxygen after 6 hours, both at 37°C in M200 (Figure 4, (a) versus (b)) and at 4°C in both solutions (Figure 4, (e), (g) versus (f), (h)). The absence of oxygen effect was also evident after 24h incubation (Supplementary Figure 2).

Study of microtubule alterations revealed a similar conclusion regarding the absence of influence for oxygen supply. As shown in Figure 5, restauration of oxygen to the cell does not alter the phenotype.

The same observations were performed after 24h incubation, at which time a similar lack of involvement of oxygen could be observed (Supplementary Figures 2 and 3).

### 2.4. Relationship between Cytoskeleton Stability and Cell Death

To evaluate if the observed alteration in cytoskeletal played a role in cell fate, we used pharmacological modulators of microfilaments and microtubules and tested their ability to improve primary endothelial cell survival after 24 hours cold hypoxic storage followed by 6 hours of regular culture conditions (mimicking reperfusion).

**Figure 6:**
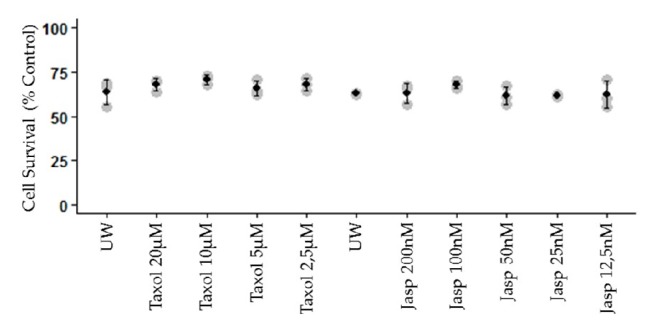
**Impact of cytoskeleton pharmaceutical stabilization on cell survival**. HAEC were cultured in hypoxia/hypothermia for 24h using either Taxol or Jasplakinolide (Jasp) and then processed for reperfusion as per the Materials and Methods. Cell survival was assessed using XTT. Statistics: ^*∗*^ p<0.05 between groups.

We first confirmed that these compounds were able to maintain cytoskeletal organization in a dose response manner (Supplementary Figure 6). Then, we observed that maintaining either microfilaments or microtubule organization throughout the 24 hours of simulated cold ischemia did not significantly improve cell survival (Figure 6) above the level of standard preservation conditions (UW, 65% survival rate).

## 3. Discussion

With the necessary evolution in donor demographics, the need for a better understanding of ischemia reperfusion becomes paramount for the establishment of more consistent and adapted protocols. In addition, characterizations of better biomarkers to quantify the quality of the organ are needed, as well as new therapeutic targets to improve viability and insure a better outcome. In this study, we investigated the impact of cold storage conditions on the cytoskeleton, a core structure of cells exposed to CS.

We used an in vitro model of hypoxia/hypothermia to mimic cold storage, using two different medium: on the one hand, the culture medium used for these cells, on the other the University of Wisconsin solution, currently one of the most used preservation solutions in transplantation [18]. Of the three types of filaments composing the cytoskeleton, only two were affected by these conditions: actin microfilaments and tubulin microtubules. Intermediate filaments were not affected.

While fluorescent images revealed a general disorganization of both filament types, the resulting effect was specific to each:

(1) Microfilaments were rapidly reduced to amorphous nodules and shortened stress fibers, leading to a loss of fibers and reorganization of the actin proteins to the cell periphery. This suggests that the cell responded to the changes in conditions with a prioritization of resources to the cell-extracellular space interface, possibly anchoring the cell.

(2) Microtubules rapidly lost their fine definition after the start of storage and reorganized near the nuclei, with loss of signal following the extension of cold ischemia time. Due to the role of microtubule in intracellular trafficking, this may imply that the cell reorganizes its transit around the nucleus to favor* de novo* protein production in order to resist organ preservation conditions.

Further probing by western blot showed that these changes appeared to stem from two different phenomena: while actin protein level remains constant overall, it appears that the ratio of organized/free actin favors the free actin portion; on the other hand, total tubulin level decreases over time, suggesting that the loss of organization and signal is due to degradation and/or production arrest.

These observations are concurrent with the bioenergetics aspect of cytoskeletal organization. Indeed, sensitivity of the cytoskeleton to hypothermia is intimately linked to the dependence of the fibers to intracellular energy: actin relies on ATP hydrolysis for its polymerization (16 ATP per 37nm) and its contractile function [19, 20], while microtubule formation requires important input of GTP (16 GTP per 8nm) [21] and vesicular transport demands 1 ATP for each 8nm traveled [22]; on the other hand, intermediate filaments have a slow turnover and their activity does not need energy; hence, the absence of influence from IR highlighted herein is logical.

Since cold storage translates into the change of 3 important parameters for the cell: temperature, environment, and oxygen tension; we endeavored to test the individual contribution of each of these factors to the observed phenotype.

UW was used herein as it remains the leading solution used worldwide and has shown to remain on par with the other solutions in terms of organ protection [23]. Unfortunately, UW is not adapted for cell preservation at 37°C; this condition was thus tested for the sake of thoroughness but could not be used to test the effect of temperature. UW was however more effective than M200 in preserving cellular integrity at 4°C, which is concordant with the rationale behind the formulation of each solution. Nevertheless, the deleterious effect of UW at 37°C did not permit us to compare the effect of different temperatures on UW stored cells.

However, use of M200 did reveal the important dichotomy between the roles played by temperature and the involvement of oxygen. Indeed, while temperature had a profound impact on both actin and tubulin organization, oxygen tension did not show any significant impact. This strongly suggests that the structures of the cytoskeleton are highly dependent on the temperature and little affected by the lack of oxygen.

One limit of our study is the limited number of temperatures tested. However, since the role of oxygen was not elucidated, the number of conditions would have been too important to permit clear conclusions to be drawn. Further studies will be necessary to elucidate the effect of varying temperature in an anoxic environment.

Finally, we tested the hypothesis that cytoskeleton protection could improve cell survival. Previous studies have shown that Taxol, preventing tubulin depolymerization, could improve viability in a canine kidney isolated tubule model [12]. In our hands, however, a similar observation could not be produced as addition of a dose range of Taxol failed to rescue cell death at the end of our cold ischemia/warm reperfusion protocol. This may be due to the difference in models (isolated tubules preserved for days versus plated endothelial cells preserved for hours) and in cell types used. Conversely, we attempted to measure the influence of actin microfilament stabilization on cell death. Here also, previous studies have shown that the use of Jasplakinolide could protect rat kidneys against cold ischemia induced apoptosis [24]. However, in our hands endothelial cells could not be protected against cell death by this compound. This may also be due to the difference in model and targeted cell type. In our endothelial cell model, cytoskeletal adaptation may be downstream from death-inducing mechanisms, such as mitochondrial dysfunction which could both influence survival and cytoskeletal organization: in this light, stabilizing the cytoskeleton would not prevent the induction of cell death. This pathway would thus not be the route of choice to establish a therapeutic strategy towards preserving the endothelium, a cell type at the center of IR and playing a central role in chronic lesion development [25].

In conclusion, we demonstrate in an* in vitro* model that, during cold storage, the endothelial cytoskeleton is deeply disorganized for both microtubules and microfilaments. These changes occur rapidly and are particularly influenced by temperature, rather than oxygenation.

## 4. Materials and Methods

### 4.1. In Vitro Model

Primary human aortic endothelial cells (HAEC) were cultured on 1% gelatin (Sigma, France) in medium 200 supplemented with 2% Low Serum Growth Factor (LSGS) and 10% fetal bovine serum (FBS) (Invitrogen, France) in a humidified atmosphere at 5% CO2 and 37°C. Cells were used at passage 5. At confluence and after synchronization, cells were subjected to several conditions: cold ischemia like conditions* via* incubation in a hermetic chamber at 4°C containing an hypoxic atmosphere: 0% O2, 5% CO2, and 95% N2 (Bactal 2 gaz, Air Liquide, France) during 24h, in University of Wisconsin solution (UW, Bridge to Life, France); several parameters were then changed to study the influence of temperature (cells kept at 37°C instead of 4°C), oxygenation (maintained in normal atmosphere rather than hypoxic), and solution (using medium 200 with 2% FBS, thereafter referred to as M200, instead of UW). At different times of the conservation period, cells were collected for analysis. Reperfusion, when testing for cell survival, was mimicked by restoring normal culture conditions.

### 4.2. Pharmacological Modulation

To modulate cytoskeleton fibers, we used Jasplakinolide (Sigma-Aldrich, St. Quentin Fallavier, France) and Taxol (Paclitaxel, Sigma), at the concentrations indicated in the figures.

### 4.3. Immunofluorescent Staining

Cells were grown on microscope glass slides, subjected to indicated conditions and fixed with paraformaldehyde then processed for immunofluochemistry staining. Actin staining was performed with phalloidin (Sigma); *α* and *β* tubulin antibody and vimentin antibody were purchased from Cell Signaling Technologies (Ozyme, Montigny-le-Bretonneux, France).

### 4.4. Cell Proliferation Assay

Cell proliferation was assessed after 6 hours of reperfusion by measurement of the mitochondrial succinate dehydrogenase activity with the XTT kit (Sigma) following the manufacturer's guidelines. Reactions were quantified by spectrophotometer (Victor3, Perkin-Elmer, France).

### 4.5. Western Blotting

Cells were grown in culture flasks. The soluble fraction of the cytoskeleton was separated from the insoluble fraction using a specific buffer (12) (1 mM MgCl2, 2 mM ethylene glycol-bis ([beta]-aminoethyl ether)-N,N,N′,N′-tetraacetic acid [EGTA], 0.5% Nonidet (P-40, Sigma, St. Louis, MO), 0.25 mg/mL aprotinin, 200 *μ*g/mL soybean trypsin inhibitor, 5 mM [epsilon]-aminocaproic acid, 1 mM benzamide, 20 mM Tris-HCl, pH 6.8 with NaOH). Each fraction was separated on a 4-15% TGX gel (Biorad, Marnes-la-Coquette, France) and transferred on a PVDF membrane. Actin staining was performed with anti actin antibody (Sigma); *α* and *β* tubulin antibody was purchased from Cell Signaling Technologies (Ozyme, Montigny-le-Bretonneux, France).

### 4.6. Statistics

Data were analyzed using NCSS software [26] and are expressed as the mean ± SD. Data normality was assessed by performing a Shapiro-Wilk W Test. Normally distributed data were analyzed with one-way ANOVA followed by pairwise t-test with pooled SD with Bonferroni correction. Nonnormally distributed data were analyzed with Kruskal-Wallis test followed by a Dunn's post hoc test. For comparisons between two groups, a Wilcoxon-Mann–Whitney test was performed. A p value < 0.05 was considered statistically significant.

## Figures and Tables

**Figure 1:**
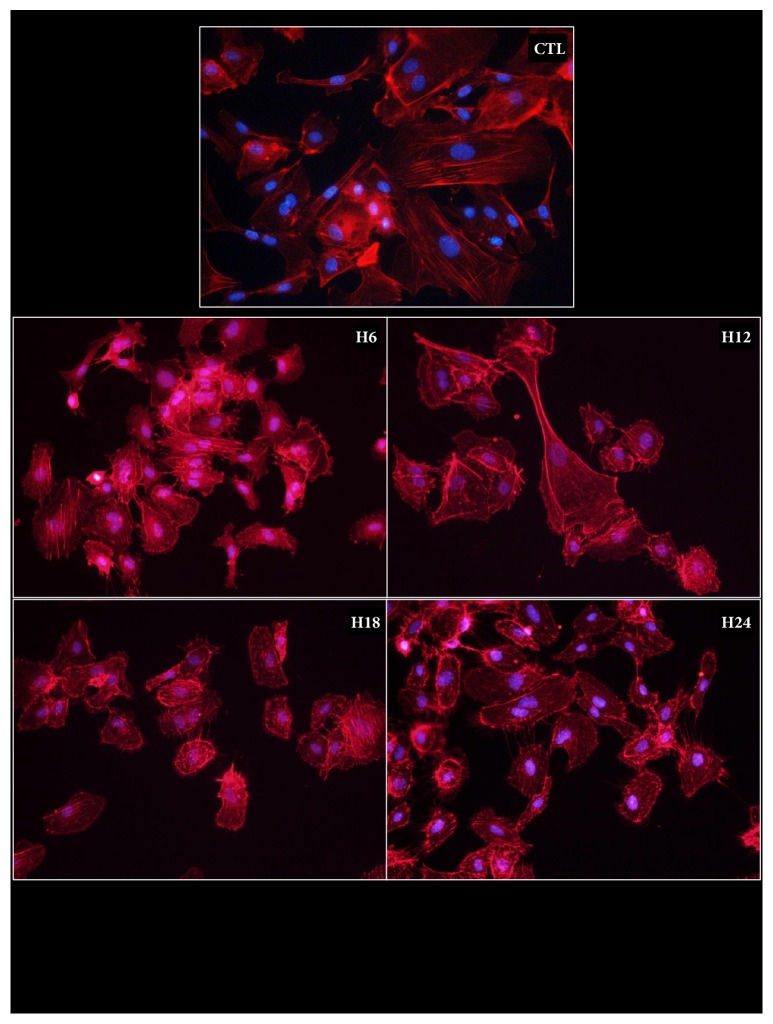
**Actin microfilament phenotype during cold ischemia**. HAEC were cultured in hypoxia/hypothermia using UW solution for different lengths of time then stained with phalloidin as per the Materials and Methods. Representative staining is shown for each duration.

**Figure 2:**
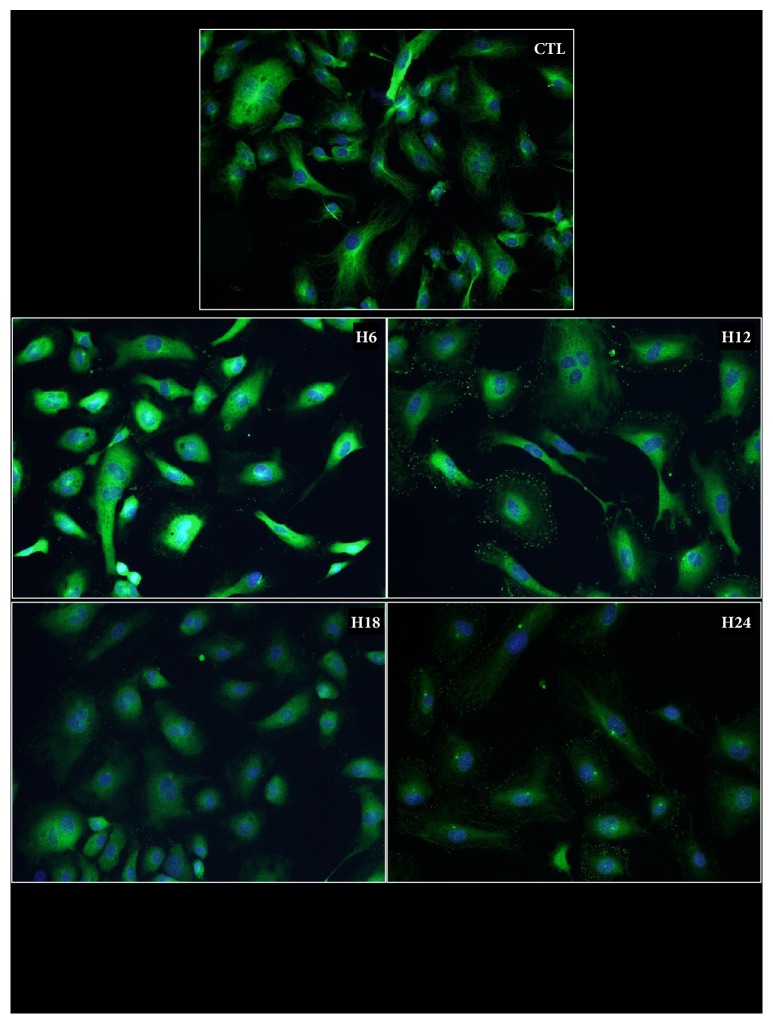
**Microtubules phenotype during cold ischemia**. HAEC were cultured in hypoxia/hypothermia using UW solution for different lengths of time and then stained with an anti *α* and *β* tubulin antibody as per the Materials and Methods. Representative staining is shown for each duration.

## Data Availability

The data used to support the findings of this study are included within the article.

## Abbreviations

MDPI: Multidisciplinary Digital Publishing Institute
DOAJ: Directory of open access journals
IRI: Ischemia reperfusion injury
HAEC: Primary human aortic endothelial cells
LSGS: Low serum growth factor
UW: University of Wisconsin solution
FBS: Fetal bovine serum
EGTA: Ethylene glycol-bis ([beta]-aminoethyl ether)-N,N,N′,N′-tetraacetic acid.

## Supplementary Materials

Supplementary 1 Supplementary Figure 1:** intermediate filament phenotype during cold ischemia**. HAEC were cultured in hypoxia/hypothermia using UW solution for different lengths of time and then stained with an anti-vimentin antibody as per the Materials and Methods. Representative staining is shown for each duration.

Supplementary 2 Supplementary Figure 2:** microfilament phenotype alteration after 6h: influence of solution, temperature, and oxygenation level**. HAEC were cultured in different conditions for 24h and then stained with phalloidin as per the Materials and Methods. Representative staining is shown for each condition.

Supplementary 3 Supplementary Figure 3:** microtubules phenotype alteration after 6h: influence of solution, temperature, and oxygenation level**. HAEC were cultured in different conditions for 24h and then stained with an anti *α* and *β* tubulin antibody as per the Materials and Methods section. Representative staining is shown for each condition.

Supplementary 4 Supplementary Figure 4:** intermediate filament phenotype alteration after 6h: influence of solution, temperature, and oxygenation level**. HAEC were cultured in different conditions for 24h and then stained with an anti-vimentin antibody as per the Materials and Methods. Representative staining is shown for each condition.

Supplementary 5 Supplementary Figure 5:** intermediate filament phenotype alteration after 24h: influence of solution, temperature, and oxygenation level**. HAEC were cultured in different conditions for 24h and then stained with an anti-vimentin antibody as per the Materials and Methods. Representative staining is shown for each condition.

Supplementary 6 Supplementary Figure 6:** confirmation of effectiveness for Taxol or Jasplakinolide**. The effect of each compound was visually verified on HAEC cultured in different conditions for 24h.** Top**: staining with falloidin showed dose effect on actin cytoskeleton stabilization, using as low dose as 50nM Jasplakinolide.** Bottom**: staining with an anti *α* and *β* tubulin showed Taxol's ability to stabilize microtubules in a dose-effect manner.
