# Supplementary figures and images for: Impact of Hypothermia and Oxygen Deprivation on the Cytoskeleton in Organ Preservation Models
Source: Biomed Res Int. 2018 Jul 16;2018:8926724. doi: 10.1155/2018/8926724 (PMC6076979; doi:10.1155/2018/8926724)

## Slide 1
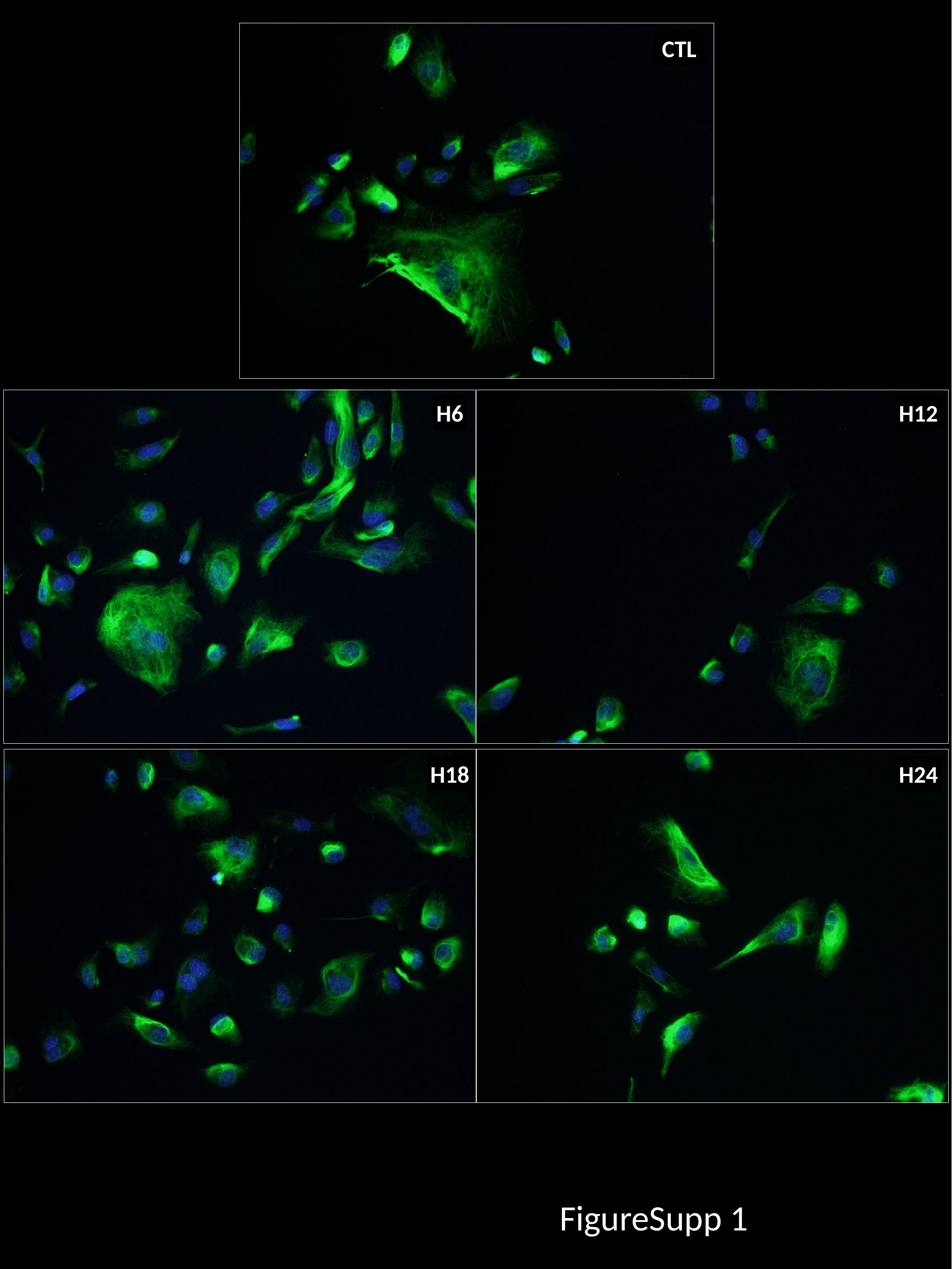

CTL
H12
H6
H18
H24
FigureSupp 1

Supplement: Supplementary 1 — Supplementary Figure 1: intermediate filament phenotype during cold ischemia. HAEC were cultured in hypoxia/hypothermia using UW solution for different lengths of time and then stained with an anti-vimentin antibody as per the Materials and Methods. Representative staining is shown for each duration. [file 8926724.f1.pptx]

## Slide 1
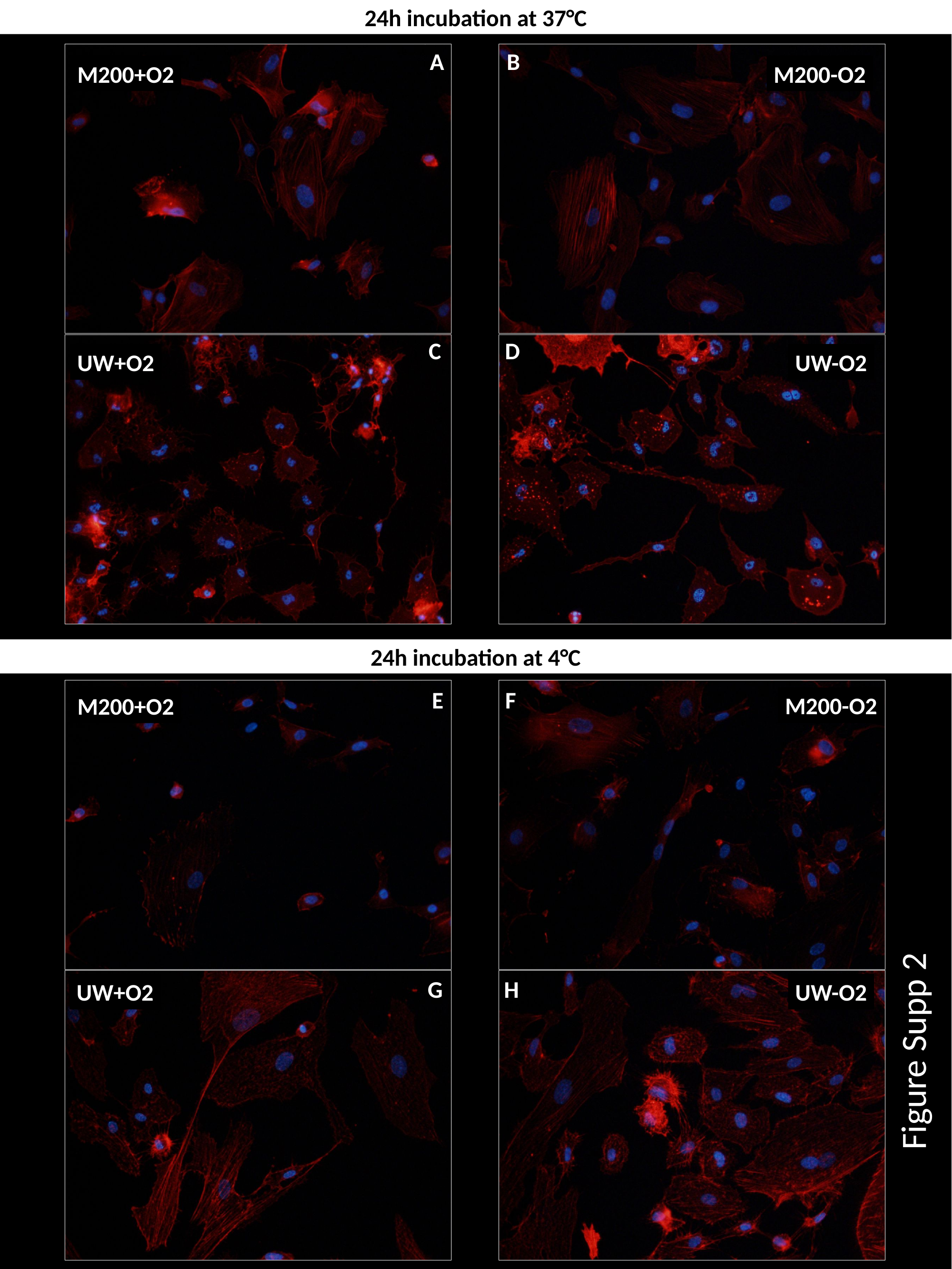

24h incubation at 37°C
A
B
M200+O2
M200-O2
C
D
UW+O2
UW-O2
24h incubation at 4°C
E
F
M200-O2
M200+O2
G
H
UW+O2
UW-O2
Figure Supp 2

Supplement: Supplementary 2 — Supplementary Figure 2: microfilament phenotype alteration after 6h: influence of solution, temperature, and oxygenation level. HAEC were cultured in different conditions for 24h and then stained with phalloidin as per the Materials and Methods. Representative staining is shown for each condition. [file 8926724.f2.pptx]

## Slide 1
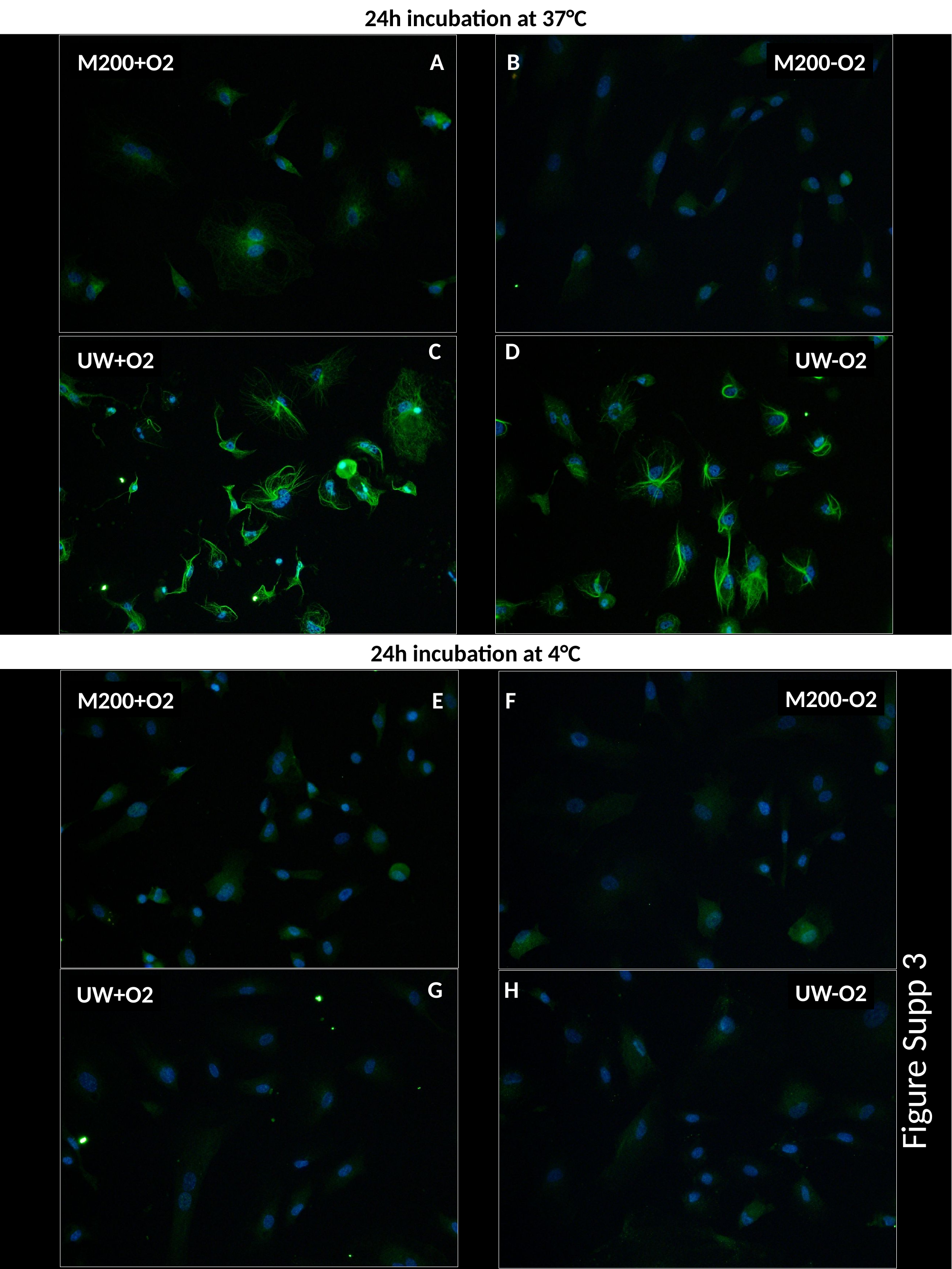

24h incubation at 37°C
A
B
M200+O2
M200-O2
C
D
UW+O2
UW-O2
24h incubation at 4°C
M200-O2
E
F
M200+O2
G
H
UW-O2
UW+O2
Figure Supp 3

Supplement: Supplementary 3 — Supplementary Figure 3: microtubules phenotype alteration after 6h: influence of solution, temperature, and oxygenation level. HAEC were cultured in different conditions for 24h and then stained with an anti α and β tubulin antibody as per the Materials and Methods section. Representative staining is shown for each condition. [file 8926724.f3.pptx]

## Slide 1
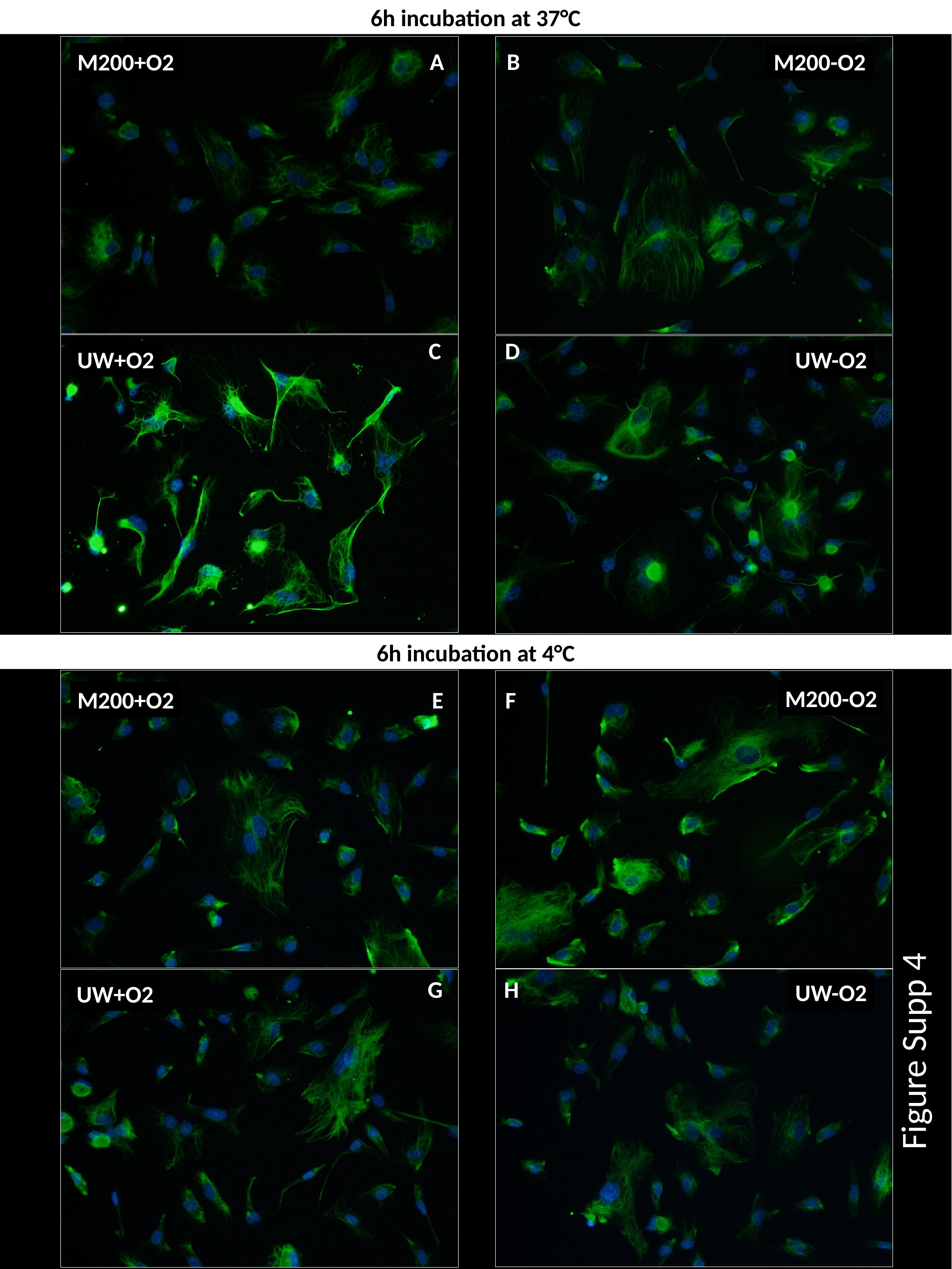

6h incubation at 37°C
A
B
M200+O2
M200-O2
C
D
UW+O2
UW-O2
6h incubation at 4°C
M200-O2
E
F
M200+O2
G
H
UW-O2
UW+O2
Figure Supp 4

Supplement: Supplementary 4 — Supplementary Figure 4: intermediate filament phenotype alteration after 6h: influence of solution, temperature, and oxygenation level. HAEC were cultured in different conditions for 24h and then stained with an anti-vimentin antibody as per the Materials and Methods. Representative staining is shown for each condition. [file 8926724.f4.pptx]

## Slide 1
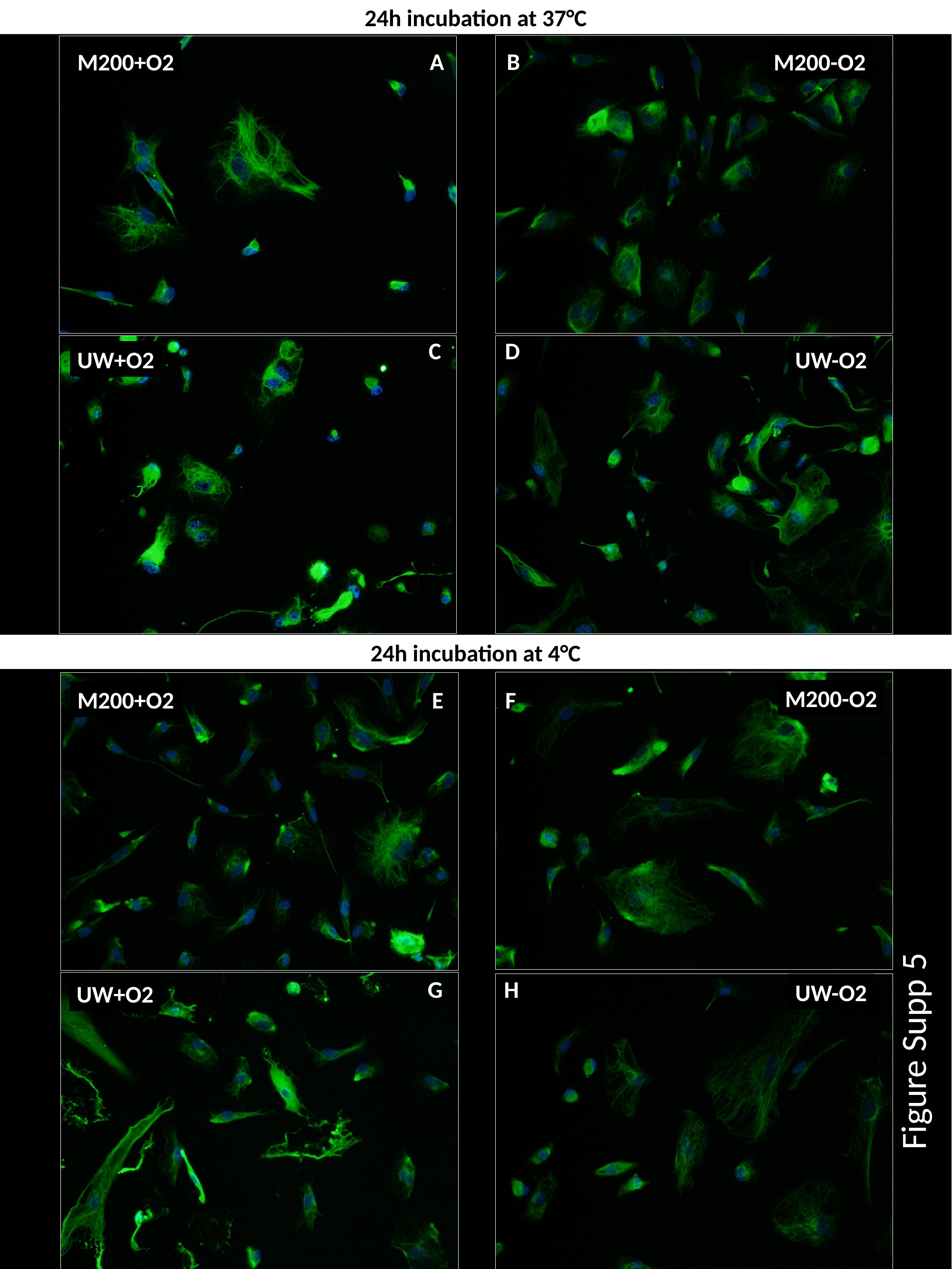

24h incubation at 37°C
A
B
M200+O2
M200-O2
C
D
UW+O2
UW-O2
24h incubation at 4°C
M200-O2
E
F
M200+O2
G
H
UW-O2
UW+O2
Figure Supp 5

Supplement: Supplementary 5 — Supplementary Figure 5: intermediate filament phenotype alteration after 24h: influence of solution, temperature, and oxygenation level. HAEC were cultured in different conditions for 24h and then stained with an anti-vimentin antibody as per the Materials and Methods. Representative staining is shown for each condition. [file 8926724.f5.pptx]
