# Supplementary material for: Impact of Hypothermia and Oxygen Deprivation on the Cytoskeleton in Organ Preservation Models
Source: Biomed Res Int. 2018 Jul 16;2018:8926724. doi: 10.1155/2018/8926724 (PMC6076979; doi:10.1155/2018/8926724)
Supplement: Supplementary 6 — Supplementary Figure 6: confirmation of effectiveness for Taxol or Jasplakinolide. The effect of each compound was visually verified on HAEC cultured in different conditions for 24h. Top: staining with falloidin showed dose effect on actin cytoskeleton stabilization, using as low dose as 50nM Jasplakinolide. Bottom: staining with an anti α and β tubulin showed Taxol's ability to stabilize microtubules in a dose-effect manner. [file 8926724.f6.pptx]

## Slide 1
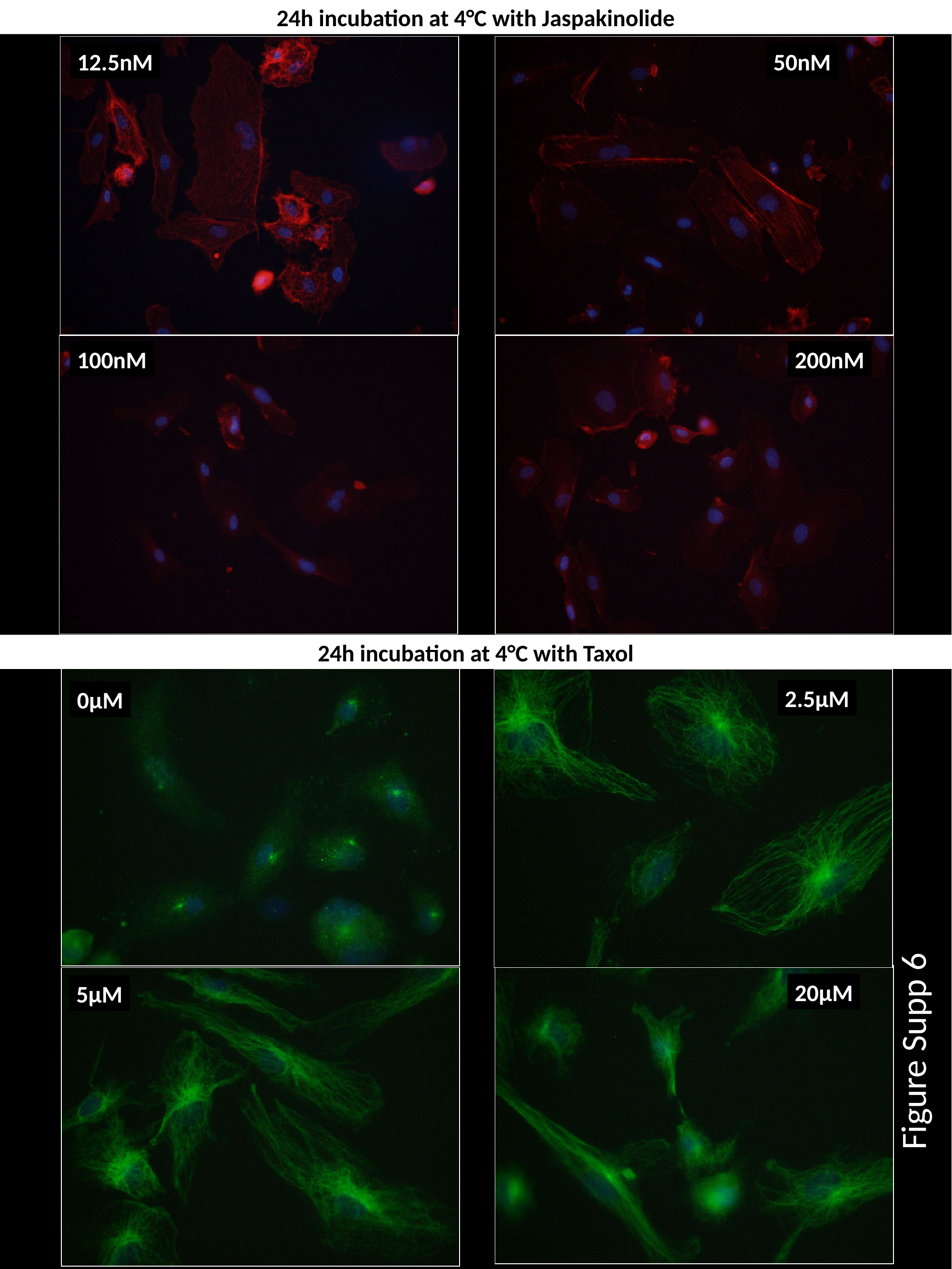

24h incubation at 4°C with Jaspakinolide
12.5nM
50nM
100nM
200nM
24h incubation at 4°C with Taxol
2.5µM
0µM
20µM
5µM
Figure Supp 6
